# Chrononutrition behavior during the COVID-19 pandemic and its relationship with body weight among college students

**DOI:** 10.3389/fnut.2023.1079069

**Published:** 2023-02-02

**Authors:** Norsham Juliana, Nur Islami Mohd Fahmi Teng, Khairunnisa Fazira Hairudin, Wan Asma Wan Abdul Fatah, Srijit Das

**Affiliations:** ^1^Faculty of Medicine and Health Sciences, Universiti Sains Islam Malaysia, Nilai, Malaysia; ^2^Faculty of Health Sciences, Universiti Teknologi MARA, Shah Alam, Malaysia; ^3^Department of Human and Clinical Anatomy, Faculty of Medicine & Health Sciences, Sultan Qaboos University, Muscat, Oman

**Keywords:** chrononutrition, chronotype, college students, young adults, overweight

## Abstract

**Introduction:**

Students in colleges are exposed to unhealthy lifestyles and poor dietary choices. They are at risk of being overweight, skipping meals, and developing eating disorders. However, there is a paucity of information on their chrononutrition behavior, which is very important, especially concerning the timing of food consumption across the day. Therefore, the present study aimed to investigate chrononutrition behavior and its potential association with body weight status among college students in Malaysia.

**Methods:**

This cross-sectional study was conducted on 409 college students aged above 18 in Malaysia. The chrononutrition behavior was assessed using the validated Chrononutrition Profile Questionnaire (CP-Q). The questionnaire was distributed using an online platform. Participants self-reported their body weight and height, and the Body Mass Index (BMI) was computed. Data were analyzed using the SPSS software.

**Results:**

A total of 409 participants were recruited, with a mean age of 21.5 ± 2.2 years. The prevalence of underweight, normal, and overweight was 24.7, 49.4, and 25.9%, respectively. The chrononutrition behavior revealed that participants ate breakfast about four times/week (mean 4.27 ± 2.43 days), and only 135 (33.0%) consumed breakfast daily. The largest meal consumed was during lunch (75.8%), and the mean of snacking after the last meal was 3.23 ± 2.01 days. The prevalence of night eating was low, and most participants (70.9) did not wake up at night to eat. The frequency, however, was significantly higher in the underweight group compared to the normal weight group (*p* < 0.05). We observed a significant association between BMI and eating window, evening latency, evening eating, and night eating. It was found that the underweight had a poor eating window (*p* < 0.01), poor evening latency (*p* < 0.01), poor evening eating (*p* < 0.01), and poor night eating (*p* < 0.05) compared to those with normal and overweight BMI groups. In contrast to predictions, poor chrononutrition behavior was more likely to predict being underweight compared to normal (*p* < 0.05).

**Conclusion:**

Underweight young adults are more likely to have poor chrononutrition behavior. The results of the present study suggest that future nutrition education should also focus on the chrononutrition behavior of college students.

## 1. Introduction

Eating habits have been a major concern among college students. They are at risk of developing unhealthy eating habits such as breakfast skipping, fast food consumption, and high sugar intake (1) that may hinder their health and psychological wellbeing (2, 3). Skipping breakfast affects the hypothalamic-pituitary function and the reproductive cycle by disturbing the central clock system, which leads to ovarian and uterine malfunction (4). The post-adolescent students’ food habits may deteriorate early in college life due to the large number of students who move in alone. The transition to college life, together with academic and lifestyle challenges, was postulated with these significant changes in their nutrition intake. Unhealthy lifestyles, including poor sleep habits, skipping food, poor eating patterns, increased alcohol intake and reduced physical activity, were also reported among college students (5). Consequently, the incidence of weight gain and increased adiposity during college life was identified in previous meta-analyses (6, 7). Nevertheless, the prevalence of being underweight among young adults and college students remains a major concern (8, 9).

While many factors related to overweight and underweight among college students have been reported, there is a paucity of information on their chrononutrition behavior. The emerging field of chrononutrition provides valuable information on managing food intake across the day. Chrononutrition is the interplay between nutrition and circadian rhythm (10). The two crucial elements are dietary components that regulate the circadian system and meal timings that synchronize misaligned molecular clocks (11). In a healthy human, the circadian rhythms play major physiological functions, including the 24-h biological cycle, behavioral, physical, and mental changes. The timing of eating is tied to the internal 24-h biological timing system (the circadian clock) and influence the metabolic process of the body due to the complex interaction between circadian biology, nutrition, and human metabolism (12). Chrononutrition behavior refers to the behavioral patterns that are likely to influence one’s chrononutrition profile, and these include (i) eating at night, (ii) time-restricted feeding, (iii) breakfast eating, (iv) the timing of the largest meal, (v) the time of evening eating, and (vi) the time between eating and sleep time (13). The chrononutrition behavior may affect an individual’s adiposity, which may be related to the physiological adaptation to sleeping and irregularities in eating following circadian times (14). Despite using less energy, individuals with more irregular eating patterns, were more likely to develop obesity and the metabolic syndrome (14).

Studies have shown that eating at different times of the day may affect one’s Body Mass Index (BMI). Inappropriate eating habits such as skipping breakfast and night eating were associated with a high BMI (15, 16). Eventually, high-calorie intake during breakfast was significantly associated with higher weight loss compared to large consumption of calories during dinner (17). It was also reported that individuals who had a high-calorie intake at later time of the day, were exposed to the risk of developing obesity. This is due to the process of lipogenesis and accumulation of adipose tissues, which tend to occur during the period of the last meal (18). Late eating may increase hunger and altered appetite-regulating hormones and altered adipose tissue gene expression favoring increased lipid storage (19).

However, the habit of skipping breakfast was also reported as a means of reducing total daily energy intake (20, 21). Indeed, it raises questions about meal timing studies such as intermittent fasting and time-restricted feeding, which indicate that skipping or delaying breakfast may reduce body weight (22, 23). The relationship between body weight and the meal timing pattern remains unclear, and it is an important research area to explore. Thus, the present study aimed to investigate the potential association of chrononutrition behavior with BMI among young adults, especially college students. To the best of our knowledge, no regional (Malaysia and Southeast Asia) studies on chrononutrition among college students has been published yet. Eating behavior of college students may carry over to later life, hence early identification related to the behavior and its effect on nutritional status (BMI) should be identified and intervene earlier. Consistent with previous studies, we hypothesized to observe significant differences concerning chrononutrition patterns and body weight status.

## 2. Materials and methods

### 2.1. Subjects

A cross-sectional study of chrononutrition behavior and weight status was conducted on college students in Malaysia’s Klang Valley. The recruitment was conducted *via* social media, and electronic forms of questionnaires were distributed. Inclusion criteria were college students aged 18−35 years, literate in the English language, and having access to the internet. Those individuals with a known diagnosis of sleep disorder were excluded. The sample size was derived using OpenEpi calculated software, with 95% confidents interval and 80% power of the study, and yielded at least 385 participants.

This study was approved by the UiTM Ethics Research Committee [REC/06/2021 (UG/MR/589)] and digital informed consent was obtained from all participants. Data on body weight and height were self-reported, and guidelines on how to measure weight and height correctly were included in the instructions. Data collection was performed in the third quarter of 2021, when Malaysia was still in the COVID-19 pandemic but moving to the endemic transition phase. The lockdown is over, and there was no restriction on going out. During this period, college students still adapted to online learning and physical attendance at the institution was not compulsory.

### 2.2. Instruments

The Chrononutrition Profile Questionnaire (CP-Q) was used to determine the chrononutrition behavior (13). Data from CP-Q measures the chrononutrition pattern on typical work/school as well as free days and can compute eating misalignment. Regarding the present study, we measured six chrononutrition behaviors: Breakfast skipping, largest meal, evening eating, evening latency, night eating, and eating window. Breakfast skipping refers to days per week during which individuals skip breakfast. The largest meal refers to the meal in which the largest number of calories are consumed. Evening eating refers to the last eating event, while evening latency refers to the duration of time between an individual’s last eating event and sleep onset. Night eating refers to one or more days per week during which individuals wake up in the night to eat. Lastly, the term “eating window” refers to the duration of time between one’s first and last eating events of the day. Results were further scored into three; good, fair, and poor behavior, a scoring cut-off from a study by Engwall (24).

Body Mass Index was computed by dividing weight (kg) by height squared (m^2^). It was then further classified as: <18.5 kg/m2 underweight, 18.5−24.9 kg/m2 normal, and >25 overweight (25).

### 2.3. Statistical analysis

All analyses were performed using SPSS statistical software version 22.0 (SPSS Inc., Chicago, IL, USA). The participant’s background including sex, age, height, weight, and BMI were reported descriptively in mean (standard deviation) or frequency. Normality tests were conducted before parametric analyses. One-way ANOVA was used to investigate differences in mean intake (days) of breakfast, snacking after last meals, and night eating between the different BMI groups. Pearson chi-squared analyses were used to determine the differences between BMI and chrononutrition behaviors. All significant variables (*p* < 0.05) were then analyzed in a logistic regression model to determine the predictor of BMI.

## 3. Results

A total of 409 participants with a mean age of 21.45 ± 2.2 years completed the questionnaire. Most of the participants were females (89.5%), and almost half of them were of normal weight (49.4%). The mean BMI was 22.4 ± 5.13 kg/m^2^. A quarter of our participants were either underweight (24.7%) or overweight (25.9%) (Table 1).

**TABLE 1:** Background of participants.

Variables	Mean ± SD/frequency (*n*)
**Sex**	
Male	43 (10.5)
Female	366 (89.5)
Age (years)	21.45 ± 2.216
Height (cm)	157.95 ± 7.151
Weight (kg)	56.12 ± 13.999
Body Mass Index (kg/m^2^)	22.4 ± 5.13
Underweight (<18.5)	101 (24.7)
Normal (18.5−24.9)	202 (49.4)
Overweight (>25)	106 (25.9)

Data were presented as *n* (%)/mean ± standard deviation (SD).

As presented in Table 2, mean intake (days per week) of breakfast and snacking after the last meal showed no significant differences between BMI categories. However, we found that the underweight group significantly had a higher intake (days per week) of night eating compared to normal weight group (*p* < 0.05). The largest meal consumed by all BMI categories was during lunch (Table 3). Timing of sleep during both free days and workdays, did not show any different between BMI. However, observing the sleeping patterns of the participants revealed that half of them sleep after midnight even on workdays. During free days, the participants were more likely to sleep after midnight.

**TABLE 2:** Mean intake (days per week) of breakfast, snacking after last meal, and eating at night.

BMI/meal timing	Underweight (*n* = 101)	Normal weight (*n* = 202)	Overweight (*n* = 106)	*P*-value
Breakfast	4.30 ± 2.49	4.38 ± 2.41	4.03 ± 2.40	0.477
Snacking after the last meal	3.53 ± 2.11	3.11 ± 2.01	3.19 ± 1.86	0.253
Night eating	1.15 ± 1.81^a^	0.67 ± 1.56^b^	0.73 ± 1.48	0.044*

Data were presented as mean ± standard deviation (SD) with significant values of *p < 0.05. Significant difference between ^a^ and ^b^ by Tukey *post hoc* analysis.

**TABLE 3:** Association between largest meal of the day and timing of sleep with BMI.

BMI/largest meal and sleep	Underweight (*n* = 101)	Normal weight (*n* = 202)	Overweight (*n* = 106)	*P*-value
**Largest meal of the day**				
Breakfast	5 (5.0)	7 (3.5)	4 (3.8)	0.235
Lunch	64 (63.4)	154 (76.2)	76 (71.7)	
Dinner	32 (31.6)	41 (20.3)	26 (24.5)	
**Time of sleep**				
**Workdays**				
9 p.m.−12 a.m.	50 (49.5)	103 (51.0)	53 (50.0)	0.967
12−6 a.m.	51 (50.5)	99 (49.0)	53 (50.0)	
**Freedays**				
9 p.m.−12 a.m.	37 (36.6)	73 (36.1)	40 (37.7)	0.369
12−6 a.m.	64 (63.4)	129 (63.9)	96 (62.3)	

Data were presented as *n* (%). Analyzed using Chi-Square Test.

Chi-square analyses revealed a significant different between BMI categories and eating window, evening latency, evening eating, and night eating (*p* < 0.05) (Table 4). It was found that being underweight was associated with poor eating habits compared to being normal and overweight. Further analyses using logistic regression identified that significant predictors of being underweight included poor eating window, poor evening latency, poor evening eating, and poor night eating (Table 5). All this poor chrononutrition behavior was strongly related to being underweight.

**TABLE 4:** Association between the scoring of chrononutrition behavior with BMI.

Chrononutrition behavior	Underweight (*n* = 101)	Normal weight (*n* = 202)	Overweight (*n* = 106)	Total (*n* = 409)	*P*-value
**^a^Eating window**					
Good (≤12:00)	54 (53.5)	132 (65.3)	83 (78.3)	269 (65.8)	0.001*
Fair (12:01−14:00)	31 (30.7)	55 (27.2)	19 (17.9)	105 (25.7)	
Poor (>14:00)	16 (15.8)	15 (7.4)	4 (3.8)	35 (8.6)	
**^b^Breakfast skipping**					
Good (1 day/week or less)	40 (39.6)	79 (39.1)	34 (32.1)	153 (37.4)	0.768
Fair (2−3 days/week)	21 (20.8)	45 (22.3)	26 (24.5)	92 (22.5)	
Poor (≥4 days/week)	40 (39.6)	78 (38.6)	46 (43.4)	164 (40.1)	
**^c^Evening latency**					
Good (>6:00)	6 (5.9)	23 (11.4)	8 (7.5)	37 (9)	0.001*
Fair (2:01−6:00)	67 (66.3)	143 (70.8)	92 (86.8)	302 (73.8)	
Poor (≤2:00)	28 (27.7)	36 (17.8)	6 (5.7)	70 (17.1)	
**^d^Evening eating**					
Good (<20:00)	6 (5.9)	41 (20.3)	19 (17.9)	65 (16.1)	0.002*
Fair (20:00−22:59)	66 (65.3)	129 (63.9)	72 (67.9)	267 (65.3)	
Poor (≥23:00)	29 (28.7)	32 (15.8)	15 (14.2)	76 (18.6)	
**^e^Night eating**					
Good (1 day/week or less)	72 (71.3)	171 (84.7)	88 (83.0)	331 (80.9)	0.042*
Fair (2−3 days/week)	14 (13.9)	17 (8.4)	12 (11.3)	43 (10.5)	
Poor (≥4 days/week)	15 (14.9)	14 (6.9)	6 (5.7)	35 (8.6)	
**^f^Largest meal**					
Breakfast	5 (5.0)	7 (3.5)	4 (3.8)	16 (3.9)	0.235
Lunch	64 (63.4)	154 (76.2)	76 (71.7)	294 (71.9)	
Dinner/supper	32 (31.7)	41 (20.3)	26 (24.5)	99 (24.2)	

Data were presented as *n* (%).

Analyzed using the Chi Square Test with significant values at *p < 0.05.

^a^Eating window describes the duration between first and last eating events, presented in HH:MM format (hours per day).

^b^Breakfast skipping describes the frequency of breakfast skipping, presents in days/week.

^c^Evening latency describes the duration between last eating event and sleep onset, presents in HH:MM format (hours per day).

^d^Evening eating describes the risk of eating late in the waking day, presented in HH:MM format (time).

^e^Night eating describes frequency of night eating, presented in days/week.

^f^Largest meal describes the meal in which largest among of food is eaten. HH:MM, hour:minute. The percentages may not total 100 due to rounding.

**TABLE 5:** Predictors of being underweight or overweight.

Variables	Underweight			Overweight		
	Odds ratio	95% CI	*P*-value	Odds ratio	95% CI	*P*-value
Poor eating window	2.607	1.205−5.644	0.015*	0.424	0.136−1.322	0.139
Poor evening latency	2.981	1.069−8.312	0.037*	0.479	0.147−1.561	0.222
Poor evening eating	6.193	2.294−16.719	<0.001*	1.012	0.446−2.296	0.978
Poor night eating	2.545	1.168−5.544	0.019*	0.833	0.309−2.242	0.717

Analyzed using Logistic regression with significant values at *p < 0.05. Reference category: Normal weight.

## 4. Discussion

Chrononutrition is closely related to the individual body’s circadian rhythm; the biological clock that regulates the sleep and wake cycle. Its response is highly influenced by changes in the environment, primarily to light and darkness. It may not be forgotten that the light/dark cycle and food intake are important circadian rhythm regulators controlled by the central clock system. These oscillations that occur in the body influence physical, mental, and behavioral changes following a 24-h cycle (18). On the other hand, the peripheral clocks in each body system that controls localized physiological processes which include glucose and lipid homeostasis, hormonal secretion, the immune responses, and the digestive system is highly influenced by the nutritional intake and physical activity patterns (26). Current studies suggest that eating time highly influences body weight; specifically, eating meals late at night may impact the desynchronization of the internal biological clock. Charlot et al. (27) suggested to prioritize in matching daily application of eating time to individual’s circadian rhythms for optimal metabolic health (27). Important circadian hormones affecting body weight, namely cortisol, serotonin, melatonin, insulin, and insulin growth factor 1 (IGF-1) are highly synchronized with the biological clock. Hence, desynchronization of meal intake will further affect these hormones and is phenotypically reflected in body weight (28).

The results of the present study found that more than 24% of participants had their largest meal at night during dinner or supper. The prevalence is in accordance with the current trend worldwide seen among young adults. Swiss adults aged 18−26 years (29), US college students (30), and Turkish university students (31) were all found to have a similar trend of night eating. Despite the fact that night eating is associated with increased body weight (15), the young adults who were underweight in our sampling frame, showed a significantly higher prevalence of night eating. Zooming at other related research that found the association between obesity and night eating, the sampling frame focused on adults at the age of more than 30 years (15, 32, 33). Guentcheva et al. (34) also found that young adults with night eating problems have lower BMI compared to those without the problem. However, the difference was not statistically significant (34). Another population of young adults that was similar to the present study was the finding among Pakistan college students who were underweight and had poorer eating habits than those with normal BMI and overweight (35). Another important aspect that must be considered in a self-reporting questionnaire to assess dietary habits is the bias that may exist in the accuracy of habitual nutritional behaviors (36). Thus, this may suggest that the effect may arise later in life. To establish the cause and effect, another important factor that must be included in future studies is the duration of individual eating habits being established.

It is important to highlight that altered food intake as shown among these young adults may affect their hormonal patterns in the long run. Since the human body has the capability of adapting and compensating for changes, the changes may not be apparent at an early age. However, recent studies have started to discuss the potential ripple effects of poor chrononutrition on health at a later age. Evidence shows that insulin sensitivity is highly associated with circadian regulation; hence, the thermic effect of food is reduced in the evening. Therefore, it was suggested that blood sugar and insulin responses to carbohydrates are more exaggerated at night than during the day (37). Recent studies suggest that endometriosis, commonly manifested by dysmenorrhea, arises as a result of the modern dietary lifestyle, thus showing the evidence of gynecological disorders being closely linked to dietary practices (38, 39). It has been reported that female students who skipped breakfast had a higher incidence of dysmenorrhea (40).

Theoretically, the time frame of intake would be higher among individuals with short sleep duration because the total caloric intake is directly associated with the time spent awake (41). The regulation will be in synchrony with normal physiological adaptation to the environmental influence (42). However, no significant differences were observed in participants, eating behaviors with their sleep duration. The finding is consistent with the findings obtained among American (43) and Brazilian adults (44).

The present study found that there was a vast heterogeneity in sleeping patterns among each participant. Benham (45) reported that the COVID-19 pandemic had a significant effect on students’ sleeping patterns. The study found that students went to bed significantly later during the pandemic and there were pronounced delays in waketimes (45). Since the present study was conducted during the pandemic after the lockdown, the wide range of waking time and sleeping time shown in the study population may have been affected by the asynchronous format of their classes’ format. Additionally, during the sampling, some of the undergraduates were still having online classes, hence, eliminating the need for early waketime.

Based on Abraham et al. (1), young adults usually establish their eating habits during their years in college and the behavior often continues through adulthood. Furthermore, Das and Evans (46) highlighted that body weight is part of the barriers and promoting factors for lifestyle choices to maintain health. The best eating habits and ideal weight can only be achieved with the help of proper knowledge about nutrition, but this knowledge needs to be combined with favorable environmental conditions such as access to nutritious food and physical activity activities (47, 48). In earlier studies, it has been reported that the absence of healthy food in educational institutions was a barrier to healthy eating (49). Self-efficacy, dietary preferences, body image, conformity to friends and parents, socioeconomic position, and the accessibility of food in the community are a few of the variables that may directly or indirectly influence adolescent eating behaviors (50).

Interestingly, earlier studies have also reported the fact that adolescents and young adults also had the habit of not consuming a proper lunch until they returned home at 03.00−04.00 p.m. (49). It has been reported that individuals who receive parental encouragement and support, tend to eat healthier foods and have better eating habits (51, 52). The underweight participants are less particular in their eating time frame (46). Good eating habits may not be the main priority being emphasized by those underweight. However, they may suffer from health consequences at a later age if their eating habits continue. Result from this study opens a new gap of study that warrant serious attention. Despite plethora of publications on eating habits of university students, most studies focused on eating disorders among those who are overweight and obese (7, 53). However, cohort studies are needed to elucidate the impact of eating disorders among those who are underweight and its association with their later health impact.

We admit a few limitations in our study. The cross-sectional design employed may be insufficient to arrive at any definite conclusion. Our study had a small sample size, hence unable to generalize to all college students in Malaysia. Besides that, participation in this study is voluntary basis, hence, resulted in marked volume of women participants as compared to the men. It was earlier reported that there are large variety in eating behavior among college students with conflicting results on the differences reflected between gender (54, 55). As weight and height data were reported, it may introduce bias, which is the limitation of all self-reported surveys. However, we provided an insight into the situation happening in the younger population. It is paramount to include the underweight in any nutrition intervention as well, as they are prone to poor eating habits.

## 5. Conclusion

This study found that underweight college students have poor chrononutrition behavior as compared to the rest. The general lack of knowledge regarding healthy and timely eating among college students is a cause of concern. These population of interest tend to skip meals and develop various eating disorders. These young adults are constantly exposed to unhealthy lifestyles and poor dietary choices without knowing the consequences of such exposure. The dietary habits of this population can be duly addressed with proper education and health screening programs. There is also a need to build strong social support and a framework to promote healthy eating among college-going young adults. Future long-term research studies should be conducted to arrive at a definite conclusion.

## Data availability statement

The raw data supporting the conclusions of this article will be made available by the authors, without undue reservation.

## Ethics statement

The studies involving human participants were reviewed and approved by the Research Ethics Committee, Universiti Teknologi MARA. The patients/participants provided their written informed consent to participate in this study.

## Author contributions

NT designed the research, supervised the project, and wrote the manuscript. NJ contributed to the research design and wrote the manuscript. KH conducted the experiment and drafted the manuscript. WW conducted the experiment. SD edited and revised the manuscript. All authors contributed to the article and approved the submitted version.

## References

[1] Abraham S Noriega B Shin J. College students eating habits and knowledge of nutritional requirements. *J Nutr Hum Health.* (2018) 2:13–7.

[2] Wattick R Hagedorn R Olfert M. Relationship between diet and mental health in a young adult Appalachian college population. *Nutrients.* (2018) 10:957. 10.3390/nu10080957 30044399 PMC6115820

[3] Hong S Peltzer K. Dietary behavior, psychological well-being and mental distress among adolescents in Korea. *Child Adolesc Psychiatry Ment Health.* (2017) 11:1–12. 10.1186/s13034-017-0194-z 29209411 PMC5706161

[4] Fujiwara T Nakata R. Skipping breakfast is associated with reproductive dysfunction in post-adolescent female college students. *Appetite.* (2010) 55:714–7. 10.1016/j.appet.2010.08.005 20728489

[5] Kanikowska D Sikorska D Kuczyńska B Grzymisławski M Brȩborowicz A Witowski J. Do medical students adhere to advice regarding a healthy lifestyle? A pilot study of BMI and some aspects of lifestyle in medical students in Poland. *Adv Clin Exp Med.* (2017) 26:1391–8. 10.17219/acem/65783 29442460

[6] Fedewa M Das B Evans E Dishman R. Change in weight and adiposity in college students: a systematic review and meta-analysis. *Am J Prev Med.* (2014) 47:641–52. 10.1016/j.amepre.2014.07.035 25241201

[7] Vadeboncoeur C Townsend N Foster C. A meta-analysis of weight gain in first year university students: is freshman 15 a myth? *BMC Obesity.* (2015) 2:22. 10.1186/s40608-015-0051-7 26217537 PMC4511069

[8] Peltzer K Pengpid S Samuels T Özcan N Mantilla C Rahamefy O Prevalence of overweight/obesity and its associated factors among university students from 22 countries. *Int J Environ Res Public Health.* (2014) 11:7425–41. 25050651 10.3390/ijerph110707425 PMC4113885

[9] Abarca-Gómez L Abdeen Z Hamid Z Abu-Rmeileh N Acosta-Cazares B Acuin C Worldwide trends in body-mass index, underweight, overweight, and obesity from 1975 to 2016: a pooled analysis of 2416 population-based measurement studies in 128.9 million children, adolescents, and adults. *Lancet.* (2017) 390:2627–42. 10.1016/S0140-6736(17)32129-3 29029897 PMC5735219

[10] Tahara Y Shibata S. Chrono-biology, chrono-pharmacology, and chrono-nutrition. *J Pharmacol Sci.* (2014) 124:320–35. 10.1254/jphs.13r06cr 24572815

[11] Ahluwalia M. Chrononutrition—When We Eat Is of the Essence in Tackling Obesity. *Nutrients.* (2022) 14:5080. 10.3390/nu14235080 36501110 PMC9739590

[12] Almoosawi S Vingeliene S Gachon F Voortman T Palla L Johnston J Chronotype: implications for epidemiologic studies on chrono-nutrition and cardiometabolic health. *Adv Nutr.* (2019) 10:30–42. 10.1093/advances/nmy070 30500869 PMC6370261

[13] Veronda A Allison K Crosby R Irish L. Development, validation and reliability of the Chrononutrition Profile-Questionnaire. *Chronobiol Int.* (2020) 37:375–94. 31760843 10.1080/07420528.2019.1692349 PMC7332160

[14] Pot G Almoosawi S Stephen A. Meal irregularity and cardiometabolic consequences: results from observational and intervention studies. *Proc Nutr Soc.* (2016) 75:475–86. 10.1017/S0029665116000239 27327128

[15] Okada C Imano H Muraki I Yamada K Iso H. The association of having a late dinner or bedtime snack and skipping breakfast with overweight in Japanese women. *J Obesity.* (2019) 2019:2439571. 10.1155/2019/2439571 PMC6421799 30944735

[16] Ma X Chen Q Pu Y Guo M Jiang Z Huang W Skipping breakfast is associated with overweight and obesity: A systematic review and meta-analysis. *Obesity Res Clin Pract.* (2020) 14:1–8. 10.1016/j.orcp.2019.12.002 31918985

[17] Reid K Baron K Zee P. Meal timing influences daily caloric intake in healthy adults. *Nutr Res.* (2014) 34:930–5. 25439026 10.1016/j.nutres.2014.09.010 PMC4794259

[18] Mohd Azmi N Juliana N Mohd Fahmi Teng N Azmani S Das S Effendy N. Consequences of circadian disruption in shift workers on chrononutrition and their psychosocial well-being. *Int J Environ Res Public Health.* (2020) 17:2043. 10.3390/ijerph17062043 32204445 PMC7142532

[19] Vujović N Piron M Qian J Chellappa S Nedeltcheva A Barr D Late isocaloric eating increases hunger, decreases energy expenditure, and modifies metabolic pathways in adults with overweight and obesity. *Cell Metab.* (2022) 34:1486–98. 10.1016/j.cmet.2022.09.007 36198293 PMC10184753

[20] Levitsky D Pacanowski C. Effect of skipping breakfast on subsequent energy intake. *Physiol Behav.* (2013) 119:9–16. 23672851 10.1016/j.physbeh.2013.05.006

[21] Sievert K Hussain S Page M Wang Y Hughes H Malek M Effect of breakfast on weight and energy intake: systematic review and meta-analysis of randomised controlled trials. *BMJ.* (2019) 364:l42. 10.1136/bmj.l42 30700403 PMC6352874

[22] Cho Y Hong N Kim K Cho S Lee M Lee Y The effectiveness of intermittent fasting to reduce body mass index and glucose metabolism: a systematic review and meta-analysis. *J Clin Med.* (2019) 8:1645. 10.3390/jcm8101645 PMC6832593 31601019

[23] Pellegrini M Cioffi I Evangelista A Ponzo V Goitre I Ciccone G Effects of time-restricted feeding on body weight and metabolism. A systematic review and meta-analysis. *Rev Endocr Metab Disord.* (2020) 21:17–33. 31808043 10.1007/s11154-019-09524-w

[24] Engwall A. *Development, Validation and Reliability of the Chrononutrition Profile.* Fargo: NDSU Repository (2019).

[25] WHO. *WHO Fact Sheet (2016). Obesity and overweight.* Geneva: World Health Organization (2016).

[26] Papakonstantinou E Oikonomou C Nychas G Dimitriadis G. Effects of diet, lifestyle, chrononutrition and alternative dietary interventions on postprandial glycemia and insulin resistance. *Nutrients.* (2022) 14:823. 10.3390/nu14040823 35215472 PMC8878449

[27] Charlot A Hutt F Sabatier E Zoll J. Beneficial effects of early time-restricted feeding on metabolic diseases: importance of aligning food habits with the circadian clock. *Nutrients.* (2021) 13:1405. 10.3390/nu13051405 33921979 PMC8143522

[28] Mohd Azmi N Juliana N Azmani S Mohd Effendy N Abu I Mohd Fahmi Teng N Cortisol on circadian rhythm and its effect on cardiovascular system. *Int J Environ Res Public Health.* (2021) 18:676. 10.3390/ijerph18020676 PMC7830980 33466883

[29] Fischer S Meyer A Hermann E Tuch A Munsch S. Night eating syndrome in young adults: Delineation from other eating disorders and clinical significance. *Psychiatry Res.* (2012) 200:494–501. 10.1016/j.psychres.2012.07.028 22883837

[30] Yahia N Brown C Potter S Szymanski H Smith K Pringle L Night eating syndrome and its association with weight status, physical activity, eating habits, smoking status, and sleep patterns among college students. *Eat Weight Disord.* (2017) 22:421–33. 10.1007/s40519-017-0403-z 28573425

[31] Sevincer G Ince E Taymur I Konuk N. Night eating syndrome frequency in university students: Association with impulsivity, depression, and anxiety. *Klinik Psikofarmakoloji Bülteni-Bull Clin Psychopharmacol.* (2016) 26:238–47.

[32] Yoshida J Eguchi E Nagaoka K Ito T Ogino K. Association of night eating habits with metabolic syndrome and its components: a longitudinal study. *BMC Public Health.* (2018) 18:1366. 10.1186/s12889-018-6262-3 30537972 PMC6288903

[33] Ishida Y Yoshida D Honda T Hirakawa Y Shibata M Sakata S Influence of the accumulation of unhealthy eating habits on obesity in a general japanese population: The Hisayama study. *Nutrients.* (2020) 12:3160. 10.3390/nu12103160 33081125 PMC7602721

[34] Guentcheva I Dugas E Hanusaik N Drapeau V Sylvestre M O’Loughlin J. Depression symptoms and night eating in young adulthood. *Eat Weight Disord.* (2020) 25:1593–600. 31673988 10.1007/s40519-019-00796-4

[35] Irfan M Jabbar M Hameed S. Dietary habits and prevalence of underweight/obesity in Students of University of Gujrat, Pakistan. *J Liaquat Univ Med Health Sci.* (2019) 18:175–80.

[36] Alkazemi D. Gender differences in weight status, dietary habits, and health attitudes among college students in Kuwait: A cross-sectional study. *Nutr Health.* (2019) 25:75–84. 10.1177/0260106018817410 30554554 PMC6542002

[37] Kinsey A Eddy W Madzima T Panton L Arciero P Kim J Influence of night-time protein and carbohydrate intake on appetite and cardiometabolic risk in sedentary overweight and obese women. *Br J Nutr.* (2014) 112:320–7. 10.1017/S0007114514001068 24833598

[38] Harris H Eke A Chavarro J Missmer S. Fruit and vegetable consumption and risk of endometriosis. *Hum Reprod.* (2018) 33:715–27. 29401293 10.1093/humrep/dey014 PMC6018917

[39] Fontana R Della Torre S. The deep correlation between energy metabolism and reproduction: a view on the effects of nutrition for women fertility. *Nutrients.* (2016) 8:87. 10.3390/nu8020087 26875986 PMC4772050

[40] Fujiwara T. Skipping breakfast is associated with dysmenorrhea in young women in Japan. *Int J Food Sci Nutr.* (2003) 54:505–9. 14522696 10.1080/09637480310001622369

[41] Garcez M de Castro M Cesar C Goldbaum M Fisberg R. A chrononutrition perspective of diet quality and eating behaviors of Brazilian adolescents in associated with sleep duration. *Chronobiol Int.* (2021) 38:387–99. 10.1080/07420528.2020.1851704 33441036

[42] Chaput J. Sleep patterns, diet quality and energy balance. *Physiol Behav.* (2014) 134:86–91. 24051052 10.1016/j.physbeh.2013.09.006

[43] Kant A Graubard B. Association of self-reported sleep duration with eating behaviors of American adults: NHANES 2005–2010. *Am J Clin Nutr.* (2014) 100:938–47. 10.3945/ajcn.114.085191 25057157 PMC4135501

[44] de Castro M Garcez M Pereira J Fisberg R. Eating behaviors and dietary intake associations with self-reported sleep duration of free-living Brazilian adults. *Appetite.* (2019) 137:207–17. 10.1016/j.appet.2019.02.020 30844412

[45] Benham G. Stress and sleep in college students prior to and during the COVID-19 pandemic. *Stress Health.* (2021) 37:504–15. 33315301 10.1002/smi.3016

[46] Das B Evans E. Understanding weight management perceptions in first-year college students using the health belief model. *J Am Coll Health.* (2014) 62:488–97. 10.1080/07448481.2014.923429 24848103

[47] McArthur L Pena M Holbert D. Effects of socioeconomic status on the obesity knowledge of adolescents from six Latin American cities. *Int J Obesity.* (2001) 25:1262–8. 10.1038/sj.ijo.0801674 11477513

[48] Sichert-Hellert W Beghin L De Henauw S Grammatikaki E Hallström L Manios Y Nutritional knowledge in European adolescents: results from the HELENA (Healthy Lifestyle in Europe by Nutrition in Adolescence) study. *Public Health Nutr.* (2011) 14:2083–91. 21810282 10.1017/S1368980011001352

[49] Monge-Rojas R Garita C Sánchez M Muñoz L. Barriers to and motivators for healthful eating as perceived by rural and urban Costa Rican adolescents. *J Nutr Educ Behav.* (2005) 37:33–40. 10.1016/s1499-4046(06)60257-1 15745654

[50] Story M Neumark-Sztainer D French S. Individual and environmental influences on adolescent eating behaviors. *J Am Diet Assoc.* (2002) 102:S40–51. 11902388 10.1016/s0002-8223(02)90421-9

[51] Neumark-Sztainer D Story M Ackard D Moe J Perry C. The “family meal”: views of adolescents. *Journal of Nutrition Education.* (2000) 32:329–34.

[52] Gillman M Rifas-Shiman S Frazier A Rockett H Camargo C Jr. Field A Family dinner and diet quality among older children and adolescents. *Arch Fam Med.* (2000) 9:235. 10.1001/archfami.9.3.235 10728109

[53] Tavolacci M Ladner J Déchelotte P. Sharp increase in eating disorders among university students since the COVID-19 pandemic. *Nutrients.* (2021) 13:3415. 10.3390/nu13103415 34684416 PMC8537638

[54] Bernardo G Jomori M Fernandes A Proença R. Food intake of university students. *Rev Nutr.* (2017) 30:847–65.

[55] Wy G Mt M Ms Z As H. Differences in eating behaviors, dietary intake and body weight status between male and female Malaysian University students. *Malays J Nutr.* (2011) 17:213–28. 22303575

