# Corrigendum: Chrononutrition behavior during the COVID-19 pandemic and its relationship with body weight among college students

**DOI:** 10.3389/fnut.2023.1185154

**Published:** 2023-04-14

**Authors:** Norsham Juliana, Nur Islami Mohd Fahmi Teng, Khairunnisa Fazira Hairudin, Wan Asma Wan Abdul Fatah, Srijit Das

**Affiliations:** ^1^Faculty of Medicine and Health Sciences, Universiti Sains Islam Malaysia, Nilai, Malaysia; ^2^Faculty of Health Sciences, Universiti Teknologi MARA, Shah Alam, Malaysia; ^3^Department of Human and Clinical Anatomy, Faculty of Medicine & Health Sciences, Sultan Qaboos University, Muscat, Oman

**Keywords:** chrononutrition, chronotype, college students, young adults, overweight

In the published article, there was an error. A correction has been made to the **Acknowledgments** statement. This sentence previously stated:

“We gratefully acknowledge the Universiti Teknologi MARA and Universiti Sains Islam Malaysia and to thank all the responders who participated in this study.”

The corrected sentence appears below:

“We gratefully acknowledge the Universiti Teknologi MARA [600-RMC/YTR/5/3 (001/2022)] and Universiti Sains Islam Malaysia and thank all the responders who participated in this study.”

The authors apologize for this error and state that this does not change the scientific conclusions of the article in any way. The original article has been updated.

